# A comparative study of glide path instruments assessing flexibility and torsion by finite element method

**DOI:** 10.4317/jced.62820

**Published:** 2025-11-30

**Authors:** Idalia Rodríguez-Delgado, Alma Daniela Bladé-Diaz, Luis A. Reyes-Osorio, Jorge Jaime Flores-Treviño, Elizabeth Madla-Cruz, Myriam Angélica De La Garza-Ramos

**Affiliations:** 1 Universidad Autónoma de Nuevo León, Facultad de Odontología, Monterrey, Nuevo León, México; 2 Universidad Autónoma de Nuevo León, Facultad de Ingeniería Mecánica y Eléctrica, Monterrey, Nuevo León, México

## Abstract

**Background:**

This study presents a novel numerical evaluation of the bending and torsion behavior of four nickel-titanium glide path instruments, addressing a critical gap in understanding their mechanical performance under clinical conditions.

**Material and Methods:**

The evaluated instruments include the WaveOne Gold Glider with a parallelogram cross-section, the R-Pilot with an S-shaped cross-section, the ProGlider with a square cross-section, and the V Taper 2H with a triangular cross-section. Using advanced computer-aided design (CAD) software, detailed geometric models of each instrument were created, followed by numerical simulations performed in the SolidWorks finite element platform. Material properties of nickel-titanium alloys and boundary conditions were defined based on ISO 3630-1 specifications for bending and torsion tests.

**Results:**

The findings revealed significant variations in stress distribution and flexibility among the instruments. Notably, the R-Pilot demonstrated superior flexibility, being approximately 57% more flexible than the WaveOne Gold with a 28 mm deflection during bending. Conversely, the V Taper 2H exhibited the highest stress levels in bending tests. While torsional stress was comparable among V Taper 2H, ProGlider, and WaveOne Gold at approximately 500 MPa, R-Pilot showed the highest stress values under torsional loads. Additionally, distinct differences in stress distribution were observed between reciprocating and rotational glide paths.

**Conclusions:**

These insights underscore the necessity of evaluating both bending and torsion behaviors to optimize the design and clinical performance of glide path instruments.

## Introduction

**Figure 1:**
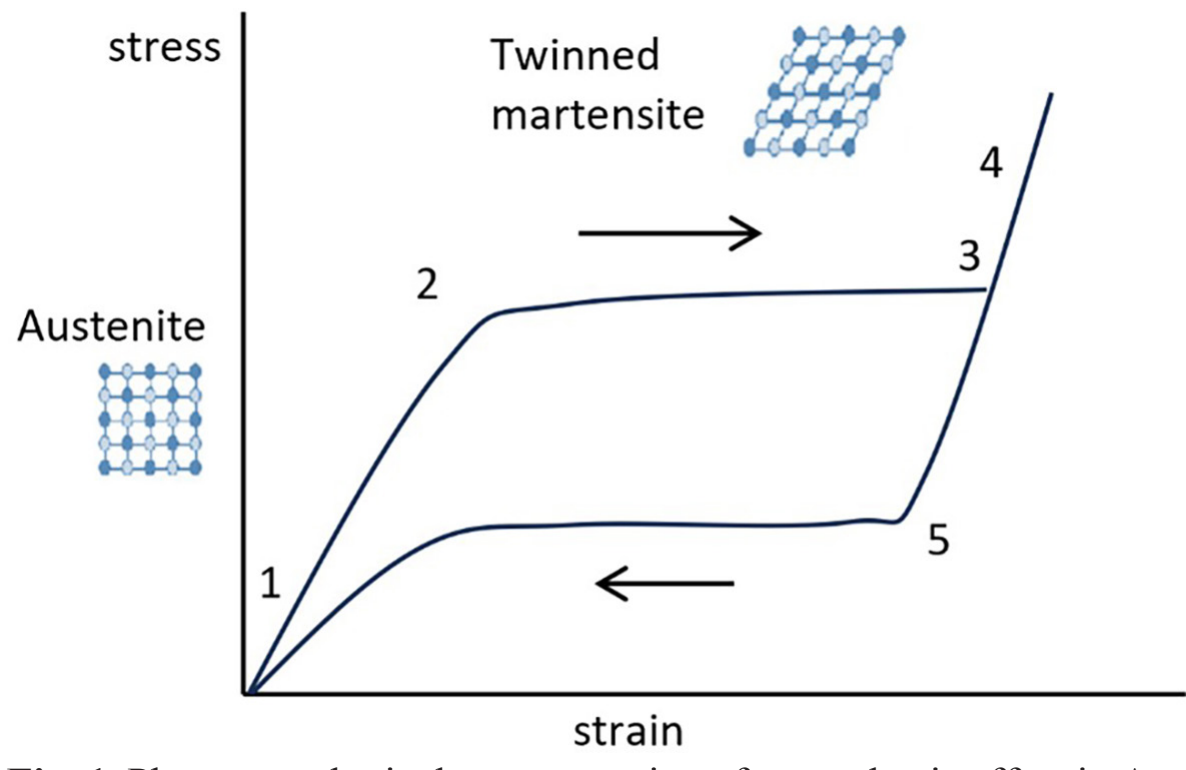
Phenomenological representation of superelastic effect in Auricchio model.

In endodontic practice, various complications can significantly impact the success rate of treatments. To address these challenges, the scientific community continuously explores innovative materials, techniques, equipment, and structural designs. Among these advancements, the glide path stands out as a critical clinical procedure. It reshapes the root canal from its coronal orifice to its apical foramen, reducing shaping errors and enhancing the safety of mechanized instrumentation. By minimizing the risk of iatrogenic complications and instrument fractures, the glide path plays a vital role in improving treatment outcomes (1). In standard endodontic procedures, the initial step is glide path preparation, which establishes a smooth and reproducible pathway to the canal's apical region. This process, typically performed with manual or rotary instruments, ensures the canal is navigable and prepared for further enlargement without complications. Following this, chemical-mechanical preparation is undertaken, combining irrigation and mechanical instrumentation to clean and shape the canal system. By performing glide path preparation first, procedural errors such as canal transportation, instrument fracture, or blockages can be effectively minimized (2). Chemical and mechanical preparation work synergistically to ensure effective root canal cleaning and disinfection. While mechanical preparation alters the root canal system by directly removing bacteria and their nutrient sources, it also enhances the penetration of chemical agents such as sodium hypochlorite [NaOCl] and ethylenediaminetetraacetic acid [EDTA] into deeper canal regions. These irrigants play a vital role in the disinfection process by fulfilling several ideal characteristics: NaOCl dissolves organic substances, EDTA removes inorganic debris, and both exhibit antimicrobial activity. Additionally, they act as lubricants for instruments and root canals, facilitating smoother procedures, while maintaining low cytotoxicity to protect surrounding tissues (3). Glide path instruments with different geometrical designs using Nickel-Titanium alloys [NiTi] were introduced to improve, innovate, reduce risks and complications. NiTi alloys are superelastic materials used for endodontic instruments (4). NiTi shape memory materials present hysteresis effects that include the dependence of the state of the system on its history. Hysteresis models, also known as constitutive models, describe the effects of superelasticity, shape memory and plasticity. In the superelastic case, the model developed by Auricchio considers different material properties in austenite and martensite phase (5). The model is developed for both one-dimensional and three-dimensional applications. The phenomenological representation of superelastic effect is shown in Fig. 1 where the elastic module of austenite is defined as 1, the stress at martensite start is in position 2, the stress of martensite final is 3, elastic modulus of martensite is 4, the stress of austenite starts is defined in point 5.

1

The high flexibility of NiTi instruments arises from the alloy's low tensile modulus of elasticity and shear modulus, which are significantly lower than those of stainless steel. However, this increased flexibility comes at the cost of lower tensile strength, making NiTi more prone to fracture under lower loads compared to stainless steel. This property influences the fracture prevalence (5). Fracture of NiTi endodontic instruments remains a problem in clinical practice (6 - 8). Fracture of endodontic files could occur in two circumstances: fracture due to torsion and bending (9). Torsion fracture occurs when the tip or any part of the instrument is blocked or bound in some part of the root canal while the shaft continues to rotate; the instrument exceeds the elastic limit of the metal and shows plastic deformation followed by fracture. This high stress is not clinically important in straight canals, where resistance to dentin removal is low; However, in curved and calcified canals, this resistance is high, and the instrument can become blocked near the tip (8 , 9). The maximum torque increases with a larger diameter of the instrument, while the flexibility decreases (10 , 11). In bending fracture, the instrument is deflected until a fracture occurs at the point of maximum flexion (8), the resistance to cyclic fatigue decreases as the maximum tensile strain amplitude on the surface of the instrument increases, which occurs at the maximum bending point during the formation of a curved root canal (10). NiTi rotary or reciprocating files have been shown to be faster and produce fewer procedural accidents. The existing literature on the mechanical performance of NiTi glide path instruments remains limited, yet several studies have explored the influence of geometric characteristics on the mechanical response of NiTi files. For example, Roda-Cassanova et al. evaluated eight rotary files by finite element method. It was reported that files with square cross section presented twice the stiffness of those with triangular cross section (12). Silva et al. observed differences in maximum torsional force that favored reciprocating files, highlighting the impact of blue thermal treatment on the torsional resistance of M-Wire Reciproc files (13). Recently, Chien et al. reported a complete review of finite element of rotary NiTi endodontic instruments (14). The use of advanced computational methods in place of large physical samples is justified by the ability of FEA to account for design-specific parameters, such as cross-sectional geometry and material properties, under idealized test conditions. A recurring theme across the literature is the significant influence of NiTi instrument design, chemical composition, and thermomechanical manufacturing processes on mechanical behavior (15). Specifically, cross-sectional configuration plays a decisive role in torsional resistance and stress distribution (16). Even instruments with the same cross-sectional design may exhibit different fracture resistances, underscoring the complexity of correlating design features with mechanical performance. These insights collectively highlight the need for further investigations to clarify these inconsistencies and refine our understanding of glide path instrument behavior under clinical conditions. In this work, four glide path files, WaveOne Gold Glider [Dentsply Sirona; Ballaigues, Switzerland] with a parallelogram cross-section, reciprocating glide path instrument from a thermomechanically treated alloy called Gold, it is manually performed by heating the instrument and then slowly cooling it, which improves the elasticity of the file; this post-manufacturing thermal process produces an instrument with superelastic NiTi metal properties that give the file a gold finish with improved mechanical characteristics (17). The ProGlider [Dentsply Sirona; Ballaigues, Switzerland] with a square cross-section is manufactured using a heat-treated M-Wire NiTi alloy to enhance flexibility and cyclic fatigue resistance (18). R-Pilot [VDW; Munich, Germany] It was the first reciprocating glide path instrument to be introduced to the market, it is manufactured from a NiTi M-Wire alloy with an S-shaped cross-section (19) and V-Glide Path 2H [SS White; Lakewood, NJ] with a triangular cross-section, is manufactured with CM-Wire alloy, which allows the file to remain flexible, even in the most curved channels (20). The four glide path files were evaluated for torsion and flexion resistance using a finite element procedure based on the ISO 3630-1 specification (21). NiTi glide path instruments are essential in modern endodontic procedures, yet their mechanical performance under clinical conditions, particularly in terms of bending and torsion behaviors, remains inadequately understood. Addressing this knowledge gap is critical for optimizing instrument design and improving clinical outcomes. This study focuses on evaluating the stress distribution and flexibility of four widely used glide path instruments, each featuring distinct cross-sectional geometries. By analyzing how these design variations influence mechanical behavior, the research aims to elucidate their implications for clinical performance and durability. The central hypothesis is that cross-sectional geometry and material properties significantly impact the bending and torsion behaviors of these instruments, affecting their flexibility, stress distribution, and overall functionality during use. Insights gained from this work will contribute to the development of more effective and reliable glide path instruments, enhancing their performance in endodontic practice.

## Material and Methods

**Figure 2:**
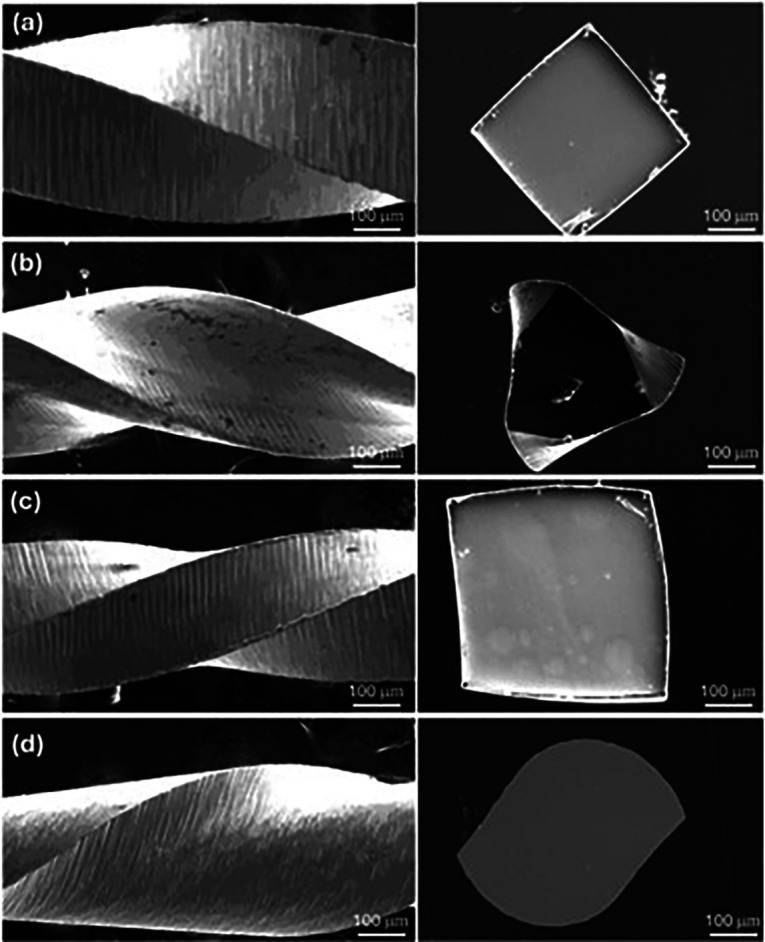
Geometrical characteristics of glide path instruments by scanning electron microscope: a] ProGlider, b] V Taper 2H, c] WaveOne Gold and d] R-Pilot.

1. NiTi instruments evaluated. The mechanical properties of four NiTi glide path instruments [WaveOne Gold Glider, R-Pilot, ProGlider, and V Taper 2H] were evaluated through bending and torsion tests following ISO 3630-1 specifications. For the bending test, each instrument was clamped at 3 mm from the tip, and a controlled force was applied to produce a standardized angular deflection, allowing the assessment of flexibility and stress distribution. For the torsion test, the instruments were secured at a specified length, and a rotational force was applied at a constant speed, recording the maximum equivalent stress. These tests were conducted using the SolidWorks finite element platform to simulate clinical conditions accurately. Boundary conditions were defined based on ISO standards, ensuring reproducibility and comparability. These evaluations were designed to assess the instruments' ability to withstand mechanical stresses encountered during endodontic procedures, providing insights into their clinical performance and durability. For the evaluation of geometrical characteristics of the glide path instruments, samples of each instrument were transversely sectioned and subsequently encapsulated in epoxy resin to ensure structural stability during preparation. They were polished to a mirror-like finish using diamond paste to enhance surface quality and facilitate detailed observation. Metallographic preparation was conducted in accordance with the ASTM E3 standard to ensure consistency and reliability in the analysis. Prior to imaging under the JSM-6510LV scanning electron microscope [JEOL Ltd, Tokyo, Japan], a thin conductive coating was applied to the samples using a Quorum 150R ES gold sputter coater, minimizing charging effects and improving image resolution. Fig. 2 shows the geometrical characteristics of the glide path instruments at different points along the file surface.

2

**Figure 3:**
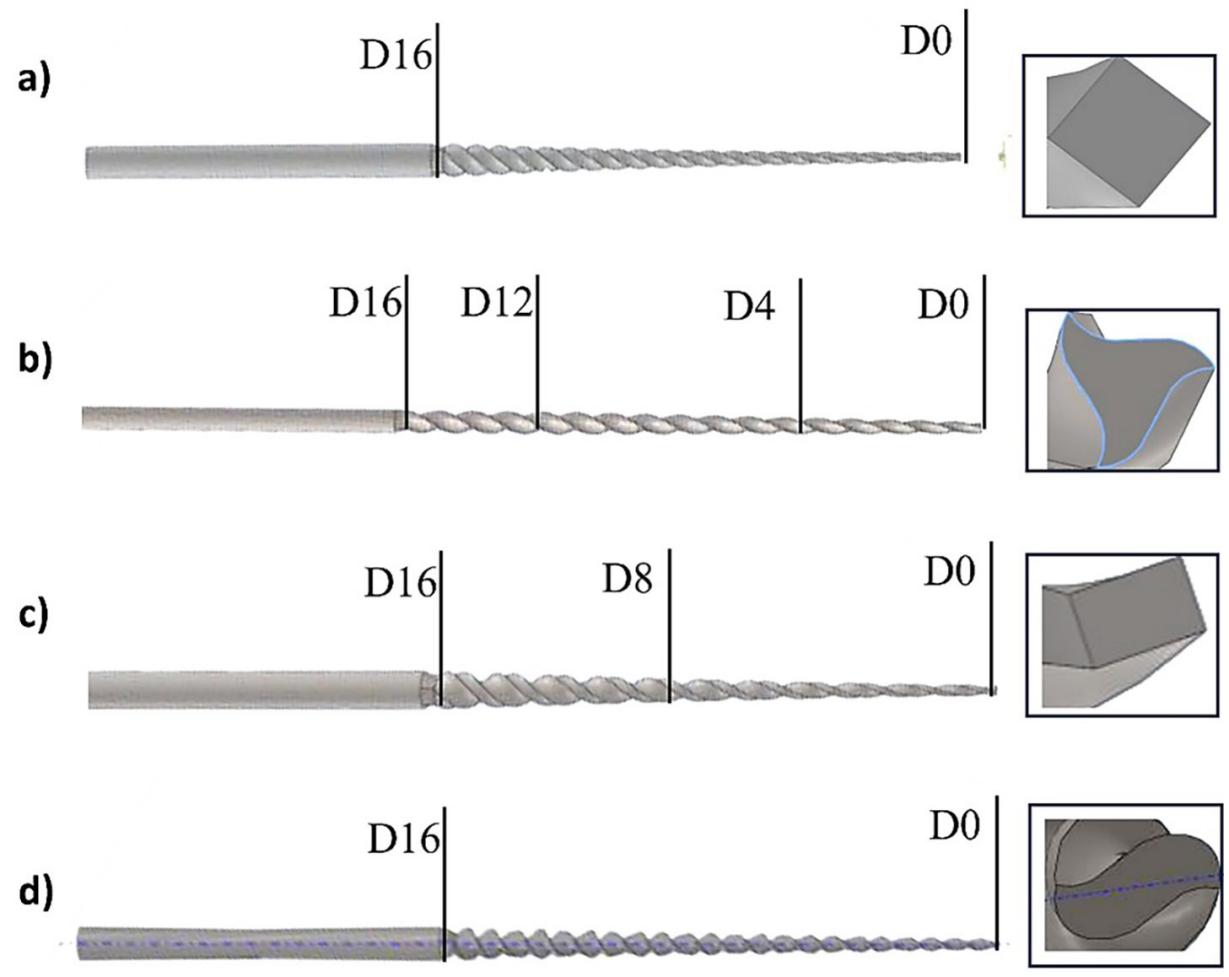
Glide path instruments: a] ProGlider, b] V Taper 2H, c] WaveOne Gold and d] R-Pilot.

The ProGlider file from the Dentsply Sirona company [Ballaigues, Switzerland] presents a diameter of 0.16 mm and a taper of 0.02 at the tip of the file (22 , 23). V-Taper 2H by SS White [Lakewood, NJ], presents a variable taper which helps preserve the dentin in the entire pericervical area starting at D0 with 0.17mm and a taper of 4% until D4, then, from D5 to D12 it presents a taper of 2%. From D13 it changes at a taper of 0%, ending in D16 at 49mm (24). WaveOne Gold by Dentsply Sirona [Ballaigues, Switzerland] has a parallelogram horizontal cross section with two cutting edges and a semi-active tip with a cone angle and a diameter of 0.15 mm at D0, it has a variable taper from 2% to 6%, resulting in a diameter at D8 of 0.41 mm and in D16 of 0.85 mm. (25 - 27). Finally, R-Pilot from the VDW company [Munich, Germany] presents a tip diameter of 0.125 mm, a taper of 4% and an S-shaped cross section (28 - 30). Fig. 3 detailed the four glide path instruments utilized in numerical study.

3

2. Numerical model. 3D models were created using SolidWorks® 2016 computer-aided design [CAD] software [Dassault Systèmes, SolidWorks Corp., Concord, MA, USA]. Subsequently, all models were transferred to the simulation library of the same program to carry out the torsion and flexion analyses. The superelastic properties of NiTi alloy are described in Table 1.

Table 1

All endodontic instruments were finely meshed, taking a minimum element of 0.078 mm, due to the geometrical characteristics of instruments and stress concentration regions of interest. Table 2 shows the number of elements and nodes for the instruments evaluated.

Table 2

**Figure 4:**
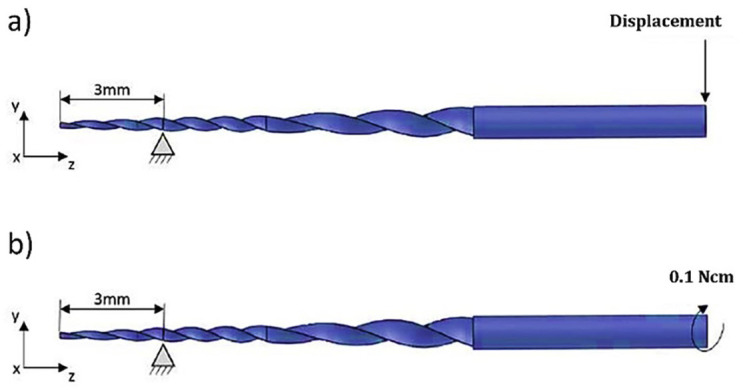
Boundary conditions for glide instruments: a] bending test and b] torsion test.

The boundary conditions for the endodontic instruments were based on ISO 3630-1 specifications. For the bending strength test, the file was kept 3 mm from the tip, avoiding any displacement in the x, y, and z axes. The axis was deviated up to a 45° inclination. This procedure was performed in the x and y directions, considering possible differences resulting from bending orientation. For the torsion resistance test, the endodontic instrument was held 3 mm from the tip and a clockwise torque of 0.1 N cm was applied. Applying a torque instead of a fixed angular deflection brings the test closer to what occurs in the clinic procedure. Fig. 4 shows the boundary conditions considered for bending and torsion tests.

4

## Results

**Figure 5:**
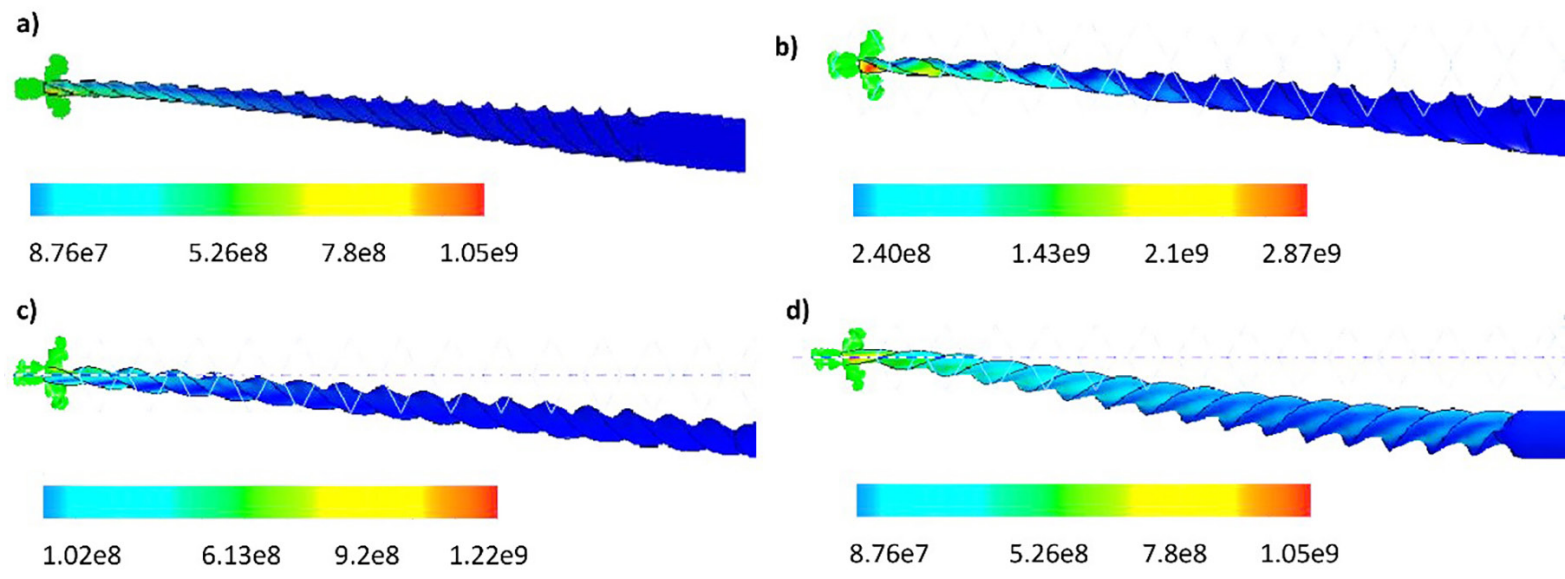
Numerical results of von Mises stress [Pa] during bending test: a] ProGlider, b] V Taper 2H, c] WaveOne Gold Glider and d] R-Pilot.

1. Bending test Fig. 5 shows the numerical results of bending test of four instruments up to 45°.

5

**Figure 6:**
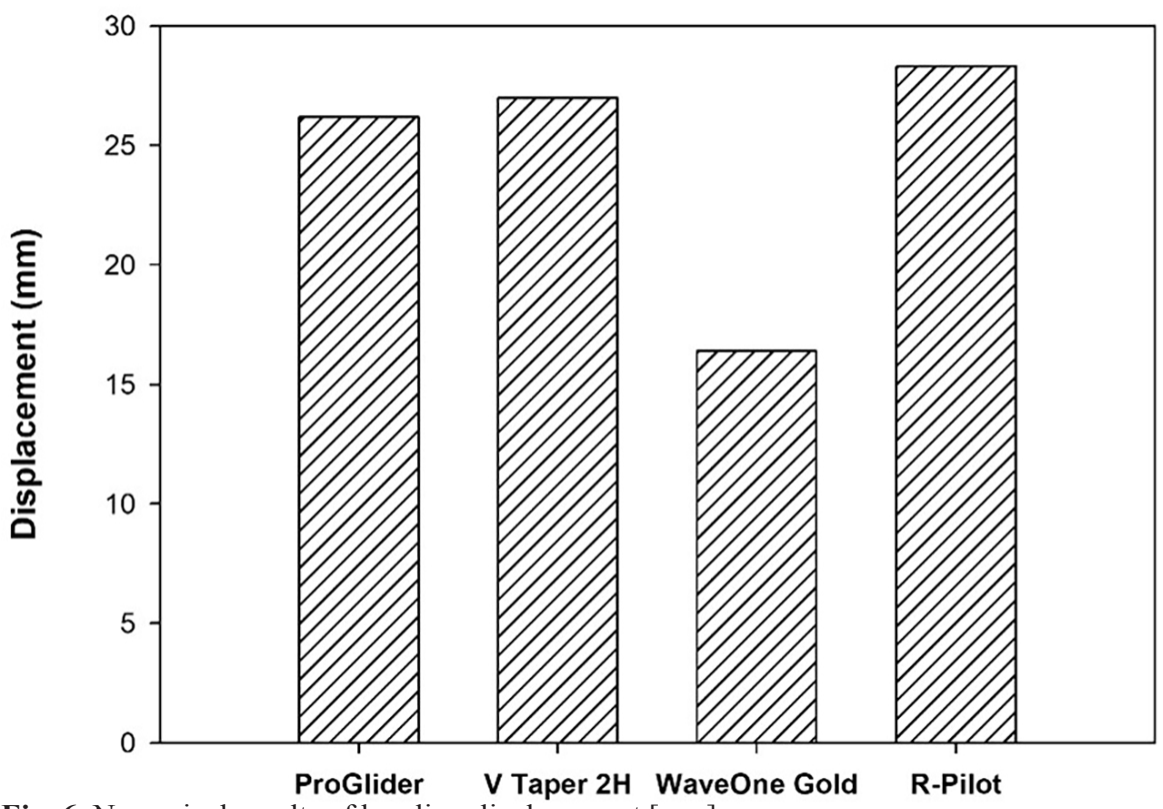
Numerical results of bending displacement [mm].

The flexibility of files can be considered as the moment required to bend the instruments without producing permanent deformation. Regarding the evaluation of flexion of the four instruments, V-Taper 2H had the highest resistance, followed by WaveOne Gold Glider. The lowest resistance was detected in ProGlider and R-Pilot. V-Glidepath 2H was 2.73 times more flexural resistant than ProGlider and R-Pilot and 2.35 times more flexural resistant than WaveOne Gold Glider. Likewise, WaveOne Gold Glider was 1.16 times more resistant than ProGlider and R-Pilot. There were no statistically significant differences between R-Pilot and ProGlider. However, V-Glidepath 2H had significant differences from the rest. This finding was also observed in WaveOne Gold Glider compared to the rest of the instruments. Regarding flexural displacement, the four instruments had statistically significant differences [p &lt;0.0001]. The instrument with the greatest flexion displacement was R-Pilot. The device with the lowest displacement was WaveOne Gold Glider, see Fig. 6.

6

**Figure 7:**
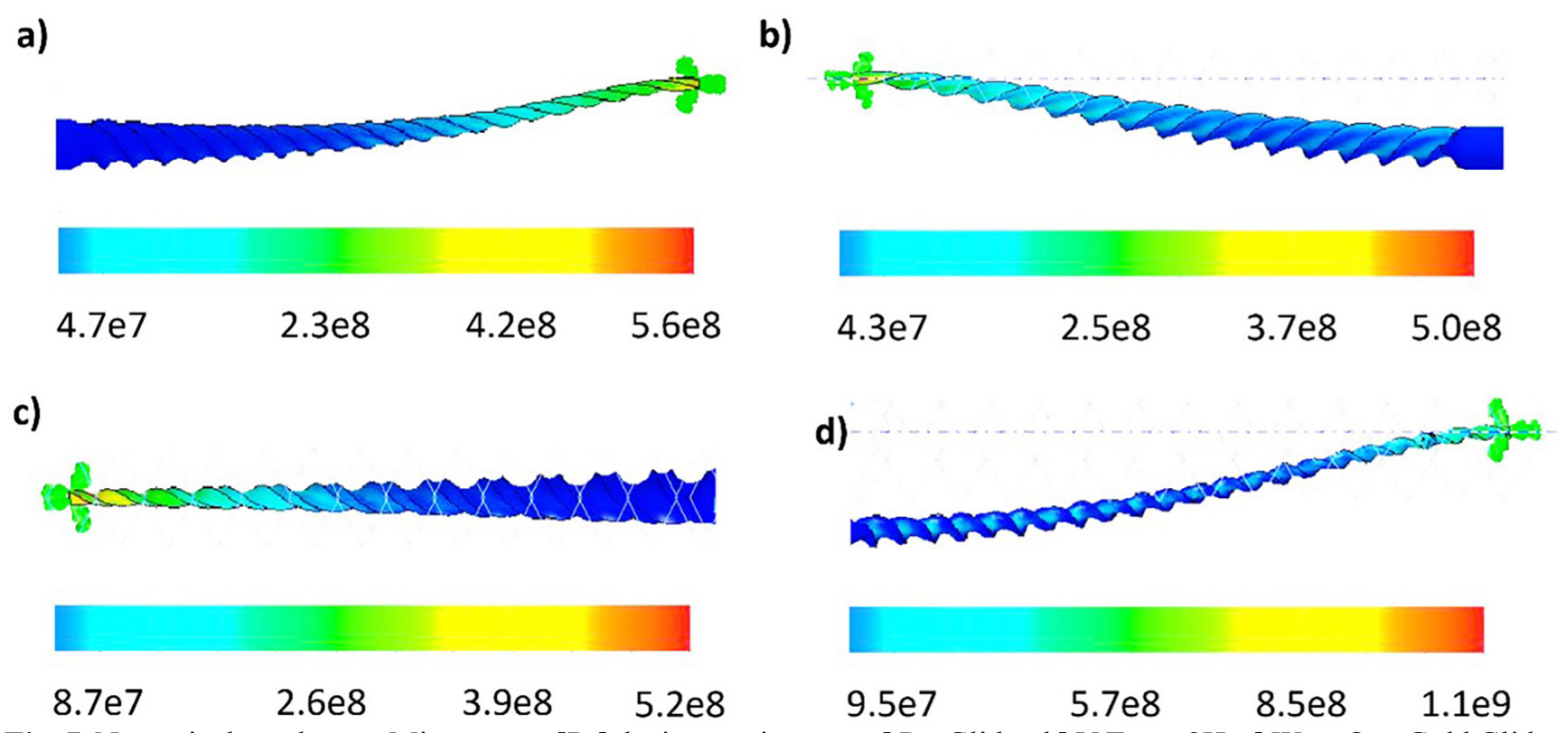
Numerical results von Mises stress [Pa] during torsion test: a] ProGlider, b] V Taper 2H, c] WaveOne Gold Glider and d] R-Pilot.

The bending displacement of R-Pilot was 1.05 times that of V-Glidepath 2H, which was 1.08 times that of ProGlider, and 1.72 times that of WaveOne Gold Glider. The flexural displacement of V-Glidepath 2H was 1.03 times that of ProGlider and 1.68 times that of WaveOne Gold Glider. Finally, the displacement of ProGlider was 1.62 times that of WaveOne Gold Glider. 2. Torsion test The maximum von Mises stress of 1,100 MPa was observed in the R-Pilot file [see Fig. 7].

7

**Figure 8:**
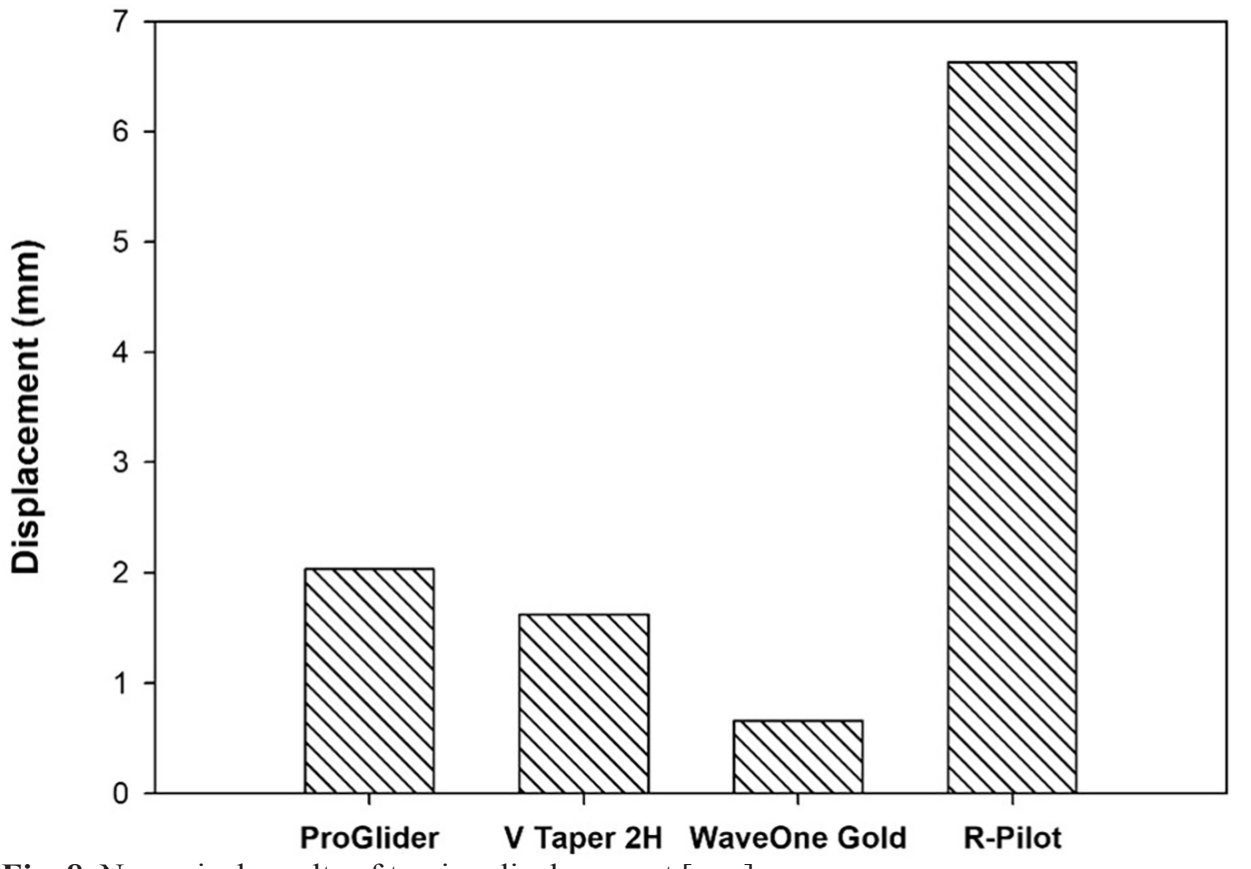
Numerical results of torsion displacement [mm].

ProGlider showed a von Mises stress of 560 MPa, while V Taper 2H exhibited the lowest torsional resistance at 500 MPa. R-Pilot torsion resistance was about 1.96 times that of ProGlider, 2.11 times that of WaveOne Gold Glider, and 2.2 times that of V-Glidepath 2H. The torsion resistance of ProGlider, the second strongest, was 1.08 times that of WaveOne Gold Glider and 1.12 times that of V-Glidepath 2H. However, WaveOne Gold Glider torsion resistance was 1.04 times higher than that of V-Glidepath 2H. The maximum displacement was observed in R-Pilot file, see Fig. 8.

8

The displacement was 3.3 times greater than ProGlider, 4.12 times greater than V Taper 2H, and 11 times greater than WaveOne Gold Glider. The ProGlider displacement was 1.25 times that of V Taper 2H and 3.33 times that of WaveOne Gold Glider file. However, the torsion displacement of V Taper 2H was 2.66 times that of WaveOne Gold Glider. The displacement observed during the torsion loading of glide path instruments is primarily a result of torsional deformation caused by the applied torque, as specified by ISO 3630-1 for torsion tests. The shape and size of the instrument's cross-section significantly influences its torsional rigidity. Instruments with smaller core diameters or less robust cross-sections [e.g., S-shaped or square] tend to deform more under the same applied torque, resulting in greater displacement. The torsion test revealed statistically significant differences among the four instruments in terms of both torsional resistance and torsional displacement. These variations highlight the distinct mechanical behaviors of each instrument, underscoring the influence of their design and material properties on their performance under torsional stress. Table 3 presents a summary of mechanical results for bending and torsion tests of different files.

Table 3

## Discussion

This study analyzed four nickel-titanium glide path instruments [WaveOne Gold glider, R-pilot, ProGlider and V Taper 2H] to determine bending and torsion properties. As main results, we found that V Taper 2H presented the highest level of stress, R-Pilot is about 57% more flexible than WaveOne Gold Glider, and for torsion R-Pilot presented the highest stress. Also, V Taper 2H, ProGlider and WaveOne Gold Glider presented very near stress values, showing a difference in stress distribution between reciprocating and rotational glide paths. The V-Taper 2H's higher flexural resistance compared to the ProGlider, R-Pilot, and WaveOne Gold Glider can be attributed to its geometric and material properties, which directly influence its mechanical behavior under bending loads. The triangular cross-section of the V-Taper 2H offers a larger moment of inertia compared to the other designs [parallelogram, S-shaped, and square cross-sections]. This larger moment of inertia increases its ability to resist deformation under flexural loads, resulting in higher flexural resistance. Unlike the R-Pilot's S-shaped or the ProGlider's square cross-section, the triangular shape distributes stress more evenly across the instrument, minimizing localized deformation and enhancing its stiffness. Although the literature on research related to this study is scarce, the findings presented here are consistent with those reported by Silva et al. (13), who observed differences in maximum torsional force favoring reciprocating files. Their study evaluated the influence of blue thermal treatment on the torsional resistance behavior of M-Wire Reciproc files [VDW, Munich, Germany]. However, these results contrast with those of Schroder et al. (31), who evaluated the effect of different glide path instruments on cyclic fatigue resistance and reported no statistical differences between G1 [manual K-file #15], G2 [Wave One Glider reciprocating instrument], and G3 [control group]. Moreover, our findings are contrary to those of Soram et al. (32), who stated that V-Taper 2H #14/03 showed superior cyclic fatigue resistance and lower ultimate strength. In contrast, Lopes et al. (33) reported that the Wave One Gold Glider [WOGG] exhibited the lowest bending resistance when compared to other glide path instruments such as the ProGlider. In their study, however, the instrument demonstrated good resistance. Additionally, Thu et al. (34) reported that ProGlider presented higher torque values when compared to TruNatomy Glider, Hyflex EDM, and Dent Craft RE glide path instruments in single- and double-curved canals, a finding similar to that of our investigation. Another important point that coincides with previous investigations is the mechanical response of NiTi files, which is related to their design, chemical composition and thermomechanical processes applied during manufacturing. In particular, the cross-sectional configuration appears to have a decisive influence on the torsional behavior and von Mises stress distribution in NiTi rotary instruments, i.e., files having the same cross-sectional design can exhibit a different resistance to fracture (32). The findings from this finite element analysis [FEA] provide insights into the performance characteristics of NiTi glide path instruments, offering valuable implications for endodontic practice. Specifically, the R-Pilot instrument demonstrated superior flexibility during bending, while also exhibiting the highest torsional stress compared to the other instruments evaluated. This suggests that R-Pilot's S-shaped cross-sectional design allows for greater deflection without permanent deformation, a critical attribute for navigating curved root canals, but it also makes the instrument more prone to torsional stress and potential fracture under clinical conditions. In contrast, the V Taper 2H exhibited the highest resistance to bending stress, indicating its rigidity when subjected to flexural forces. This could make it less suitable for negotiating complex canal anatomies but beneficial in providing strength in less curved canals. WaveOne Gold Glider and ProGlider, while showing moderate performance in both tests, may offer a balanced approach for clinicians seeking a compromise between flexibility and strength. The similar stress values observed for WaveOne Gold Glider and V Taper 2H during torsional tests suggest comparable clinical performance in rotational movements, though their differing flexibility in bending highlights the importance of matching instrument characteristics to canal geometry and clinical requirements. The difference in performance between reciprocating and rotational glide path instruments highlights the role of movement type in influencing stress distribution. Reciprocating instruments like WaveOne Gold Glider and R-Pilot may exhibit higher flexibility and lower torsional resistance, but their unique movement patterns may reduce cyclic fatigue accumulation compared to purely rotational systems. The distinct characteristics of NiTi glide path instruments enable tailored selection for specific root canal anatomies. R-Pilot is ideal for curved and complex canals due to its superior flexibility but requires careful handling to avoid torsional stress and fracture. V Taper 2H, with its rigidity and high bending resistance, is best suited for straight or minimally curved canals, where strength is prioritized over adaptability. WaveOne Gold Glider and ProGlider offer balanced performance, making them versatile options for moderately curved canals or general use, providing a compromise between flexibility and strength. Aligning instrument choice with canal complexity can improve clinical outcomes, reduce errors, and enhance instrument longevity. One of the strengths of this study lies in its use of finite element analysis to simulate clinical conditions, offering a precise and controlled environment to evaluate the mechanical properties of the glide path instruments. Additionally, the inclusion of instruments with different cross-sectional geometries and theremomecachanically treated NiTi alloys enhances the relevance of the study, as it provides a broader comparison that can be useful for clinicians when selecting appropriate tools based on canal anatomy. Thermomechanical treatment of NiTi alloy allows a change in the phase composition leading to the appearance of martensite under clinical conditions. Whilst M-Wire instruments maintain an austenitic state, CM Wire, as well as the Gold and Blue heat-treated instruments, is composed of substantial amounts of martensite. The austenitic instruments possess superelastic properties and reveal high torque values at fracture. Thus, these files are appropriate to shape straight or slightly curved root canals. martensitic instruments are more flexible with an enhanced resistance to cyclic fatigue and reveal a greater angle of rotation but lower torque at fracture (35). However, this study is not without limitations. FEA, while highly effective for simulating mechanical behavior, does not fully replicate the complex biological environment of a clinical setting, including the variability of human dentin or the influence of cyclic fatigue over extended periods of use. The study also focused solely on static bending and torsion tests, which may not fully capture the dynamic stresses experienced by instruments during actual root canal procedures. Additionally, the study did not evaluate other critical factors such as cutting efficiency or debris removal, which are essential for the overall success of endodontic treatment. Future research should build on these findings to better understand the long-term implications of instrument design on clinical outcomes. Incorporating dynamic simulations that account for cyclic fatigue could provide valuable insights into the durability and reliability of these instruments under repeated use, reflecting real-world clinical scenarios. Such studies could elucidate how stress accumulation over time affects instrument longevity and failure rates, thereby guiding the development of more resilient designs that minimize risks to patients. Additionally, clinical trials are critical to validate the in vivo relevance of these findings. Real-world testing would assess how these instruments perform across diverse patient populations and a broader spectrum of root canal anatomies, including challenging cases with curved or calcified canals. This would help determine whether the observed mechanical advantages translate to improved procedural efficiency, reduced treatment time, and lower rates of procedural errors such as canal transportation or instrument fracture. Exploring factors such as cutting efficiency and debris removal performance would further enhance the clinical relevance of these studies. Effective cutting and debris clearance are essential for maintaining apical patency, minimizing bacterial load, and ensuring thorough canal shaping. Instruments that excel in these areas may contribute to better disinfection, reduced postoperative complications, and improved long-term success rates of endodontic treatments. The limitation of finite element analyses is that they offer an approximation; they do not replace physical or clinical tests, such as the frictional stress of files against the walls of the root canal. However, in this study, the files were subjected to the same bending and torsion parameters for analysis, conditions that would not have been possible with extracted teeth. These additional investigations would provide a more holistic understanding of the relationship between instrument design and clinical performance, driving innovations that prioritize patient safety, treatment efficacy, and practitioner convenience. For clinical practice, the findings of this work suggest that instrument selection should be guided not only by the mechanical properties of the tools but also by the specific clinical scenario. Instruments like R-Pilot, which offer high flexibility, may be advantageous in treating severely curved canals, but clinicians must be cautious of its higher susceptibility to torsional stress. Conversely, instruments with higher torsional resistance, such as V Taper 2H, may be preferable in less complex canals where rigidity is beneficial. Understanding these properties can help practitioners reduce the risk of instrument fracture and optimize treatment outcomes.

## Conclusions

In this work, four glide path instruments were evaluated numerically for bending and torsion responses. The instrument most resistant to flexion was V-Glidepath 2H. On the other hand, in the torsion test, R-Pilot, an instrument that is also used with reciprocating movement, had better results. Regarding the displacement or rigidity of the instrument, in the finite element analysis, WaveOne Gold Glider had a lower displacement in the flexion and torsion tests; therefore, it is the most rigid. The differences between reciprocating and rotational glide paths highlight the importance of instrument motion in influencing mechanical performance. These insights emphasize the necessity of integrating both bending and torsion evaluations into the design process to enhance the safety, efficiency, and clinical reliability of glide path instruments. This work provides a foundation for further optimization of instrument design, contributing to improved outcomes in endodontic practice.

## Figures and Tables

**Table 1:** Mechanical behavior of superelastic NiTi alloy [16].

Parameter	Description	Magnitude
EA	Elasticity of austenite	42530 MPa
νA	Poisson ratio of austenite	0.33
EM	Elasticity of martensite	12828 MPa
νM	Poisson ratio of martensite	0.33
εL	Transformation strain	10%
[δσ/δT]L	[δσ/δT] loading	6.7
σSL	Start of transformation loading	492 MPa
σEL	End of transformation loading	630 MPa
T0	Temperature of reference	22˚C
[δσ/δT]U	[δσ/δT] unloading	6.7
σSU	Start of transformation unloading	192 MPa
σEU	End of transformation unloading	97 MPa
σEME	End of martensitic elastic regime	1200 MPa

1

**Table 2:** Mesh characteristics of instruments evaluated.

Instrument	Number of elements	Number of nodes
ProGlider	61,179	110,184
V Taper 2H	66,955	120,588
WaveOne Gold	69543	125,250
R-Pilot	56840	102,372

2

**Table 3:** Maximum Von Mises stress [MPa] and displacement [mm] of different files.

	Bending test		Torsional test	
Instrument	Maximum stress[MPa]	Displacement[mm]	Maximum stress[MPa]	Displacement[mm]
V-Glidepath 2H	2870	27	500	1.6
ProGlider	1050	26	560	2.0
R-Pilot	1050	28	1100	6.6
WaveOne Gold Glider	1220	16	520	0.6

3

## Data Availability

All data that supports reported results are included within the manuscript.
